# Region-specific effects of Scrapper on the abundance of glutamate and gamma-aminobutyric acid in the mouse brain

**DOI:** 10.1038/s41598-020-64277-w

**Published:** 2020-05-04

**Authors:** Fumihiro Eto, Shumpei Sato, Mitsutoshi Setou, Ikuko Yao

**Affiliations:** 1 Department of Optical Imaging, Institute for Medical Photonics Research, Preeminent Medical Photonics Education & Research Center, Hamamatsu University School of Medicine, 1-20-1 Handayama, Higashi-ku, Hamamatsu, Shizuoka, 431-3192 Japan; 2 Department of Cellular and Molecular Anatomy, Hamamatsu University School of Medicine, 1-20-1 Handayama, Higashi-ku, Hamamatsu, Shizuoka, 431-3192 Japan; 3 Department of Biomedical Chemistry, School of Science and Technology, Kwansei Gakuin University, 2-1 Gakuen, Sanda, Hyogo, 669-1337 Japan; 4 International Mass Imaging Center, Hamamatsu University School of Medicine, 1-20-1 Handayama, Higashi-ku, Hamamatsu, Shizuoka, 431-3192 Japan; 5 Department of Systems Molecular Anatomy, Institute for Medical Photonics Research, Preeminent Medical Photonics Education & Research Center, Hamamatsu University School of Medicine, 1-20-1 Handayama, Higashi-ku, Hamamatsu, Shizuoka, 431-3192 Japan

**Keywords:** Neuroscience, Anatomy

## Abstract

The brain consists of various areas with anatomical features. Neurons communicate with one another via excitatory or inhibitory synaptic transmission. Altered abundance of neurotransmitters, including glutamate and gamma-aminobutyric acid (GABA), in specific brain regions is closely involved in severe neurological diseases, such as schizophrenia and obsessive-compulsive disorder. SCRAPPER, a ubiquitin E3 ligase, regulates synaptic transmission. *Scrapper* gene deficiency results in defective neurotransmission due to excessive secretion of neurotransmitters. The present study employed matrix-assisted laser desorption/ionization imaging mass spectrometry to analyze the abundance of amino acid neurotransmitters in *Scrapper* knockout (SCR-KO) mice. SCR-KO mice exhibited significantly increased glutamate levels in the isocortex (CTX), corpus callosum (CC), thalamus (TH), midbrain (MB), cerebellar cortex (CBX), and caudoputamen (CP) and increased GABA levels in the CTX, CC, TH, MB, CBX and hypothalamus (HY) compared with wild-type mice. These findings indicate that Scrapper deficiency leads to upregulated glutamate and GABA levels in multiple regions. Our results show a differential, region-specific effect of Scrapper on the abundance of glutamate and GABA.

## Introduction

The brain consists of various areas with anatomical features. Neurons communicate with one another via synaptic transmission of excitation or inhibition and form a complex biological network in the brain. Neurotransmitters, including glutamate and gamma-aminobutyric acid (GABA), are highly involved in brain functions, such as cognition and movement, and disorders of neurotransmission cause various central nervous system diseases^1–4^. Using proton magnetic resonance spectroscopy, previous studies have observed glutamate-glutamine abundance in the anterior cingulate area in the brains of patients with schizophrenia^5^ or obsessive-compulsive disorder^6^. However, the mechanisms underlying the abundance of neurotransmitters in these brain regions remain unclear.

**Figure 1:**
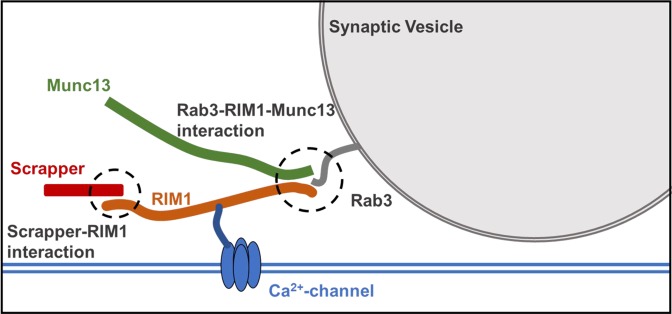
Schematic diagrams depicting the interactions between Scrapper-RIM1-Munc13 and Rab3-RIM1-Munc13.

SCRAPPER, an F-box protein encoded by *FBXL20*, is an E3 ubiquitin ligase, which is expressed in neurons. Scrapper directly binds to Rab3-interacting molecule 1 (RIM1) and mediates RIM1 degradation in neurons^7^. RIM1 binds to Munc13 in the presynaptic active zone and regulates synaptic neurotransmitter release^8–11^ (Fig. 1). Deletion of RIM1, which constitutes the scaffolding of the active synaptic zone, results in alterations of short-term plasticity and properties of evoked asynchronous release, as well as a considerable reduction in the readily releasable pool of vesicles^12^; notably, RIM1 concentrations increase in the brains of SCR-KO mice^7^. Our previous physiological studies found that deletion of Scrapper facilitated the frequency of miniature excitatory postsynaptic current (EPSC) in the hippocampal CA1^7^ and in the anterior cingulate area (limbic cortex), which are associated with pain, anxiety, and fear^13^. Furthermore, SCR-KO mice showed longer decay time of spontaneous and evoked EPSCs^13^. In fact, heterozygous SCR-KO mice exhibit reduced foot-shock-induced fear memory and abnormal social behavior^14^. Enhanced excitatory glutamatergic transmission implies that supply and/or reuptake of glutamate and the abundance of glutamate are increased in SCR-KO mice.

Glutamate and GABA distribution in the brain regions of SCR-KO mice can be assessed using matrix-assisted laser desorption/ionization (MALDI) imaging mass spectrometry (IMS). MALDI IMS can be used to assess various molecules based on their mass-to-charge ratio as well as morphological information concerning their distribution^15^. MALDI IMS further acquires and visualizes the position and intensity of neurotransmitters directly in tissue sections and provides relative degrees of changes in a region in a test group compared with a control group. The detection of small polar compounds, such as amino acid neurotransmitters, by MALDI IMS has been reported, and several on-tissue chemical derivatization methods have recently been developed, thereby enabling the *in situ* detection of amino acid neurotransmitters^16–18^.

In a previous report, we used western blotting to show that Scrapper was ubiquitously expressed throughout the brain, and used histochemical analyses to confirm that *Scrapper* mRNA and protein were highly expressed in neuronal cell-rich regions such as the hippocampus, cerebellum, and olfactory bulb^7^. However, it has not been elucidated whether Scrapper affects the abundance of glutamate throughout the entire brain. The present study thus examined the histological distribution of glutamate and GABA in SCR-KO mice using MALDI IMS and directly investigated the abundance of glutamate and GABA in each brain region to determine the effect of Scrapper.

## Results

### Derivatization and detection of glutamate and GABA in the brain sections of SCR-KO and WT mice

**Figure 2:**
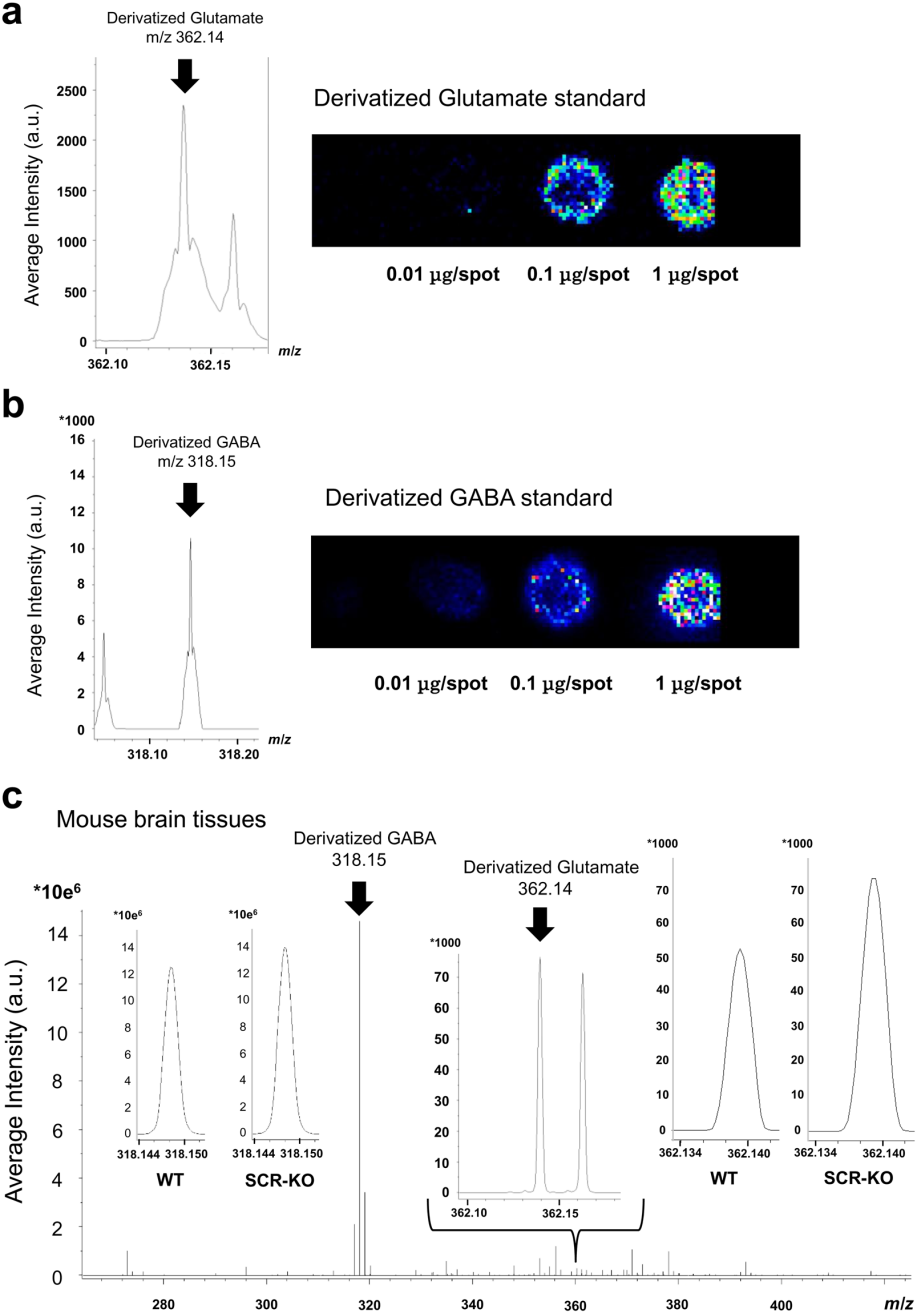
Detection of derivatized glutamate and GABA in the brain sections of SCR-KO and WT mice. Signal intensity of the derivatized standards of (**a**) glutamate (Glu) and (**b**) gamma-aminobutyric acid (GABA) on indium tin oxide-coated glass slides. (**c**) Signal intensity of derivatized Glu and GABA in brain tissues.

Standard reagents of glutamate and GABA were put on glass slides and were derivatized with 2,4-diphenyl-pyranylium tetrafluoroborate (DPP-TFB). MALDI-IMS signals were confirmed to be at *m/z* 362.136 and *m/z* 318.146. Errors in the determination of the monoisotopic mass of derivatized glutamate (Glutamate-DPP) and GABA (GABA-DPP) were both -0.003 (monoisotopic mass of Glutamate-DPP, *m/z* 362.139; monoisotopic mass of GABA-DPP, *m/z* 318.149). Signal intensities of Glutamate-DPP (Fig. 2a) and GABA-DPP (Fig. 2b) increased with increasing amounts of each standard reagent, as shown on the images of MALDI-IMS. In the brain sections treated with derivatized reagents, signals were observed at *m/z* 362.140 and *m/z* 318.148 (Fig. 2c). The error between the signal and the monoisotopic mass was 0.001.

### Glutamate increases in the isocortex, corpus callosum, caudoputamen, thalamus, midbrain, and cerebellar cortex in SCR-KO mice

**Figure 3:**
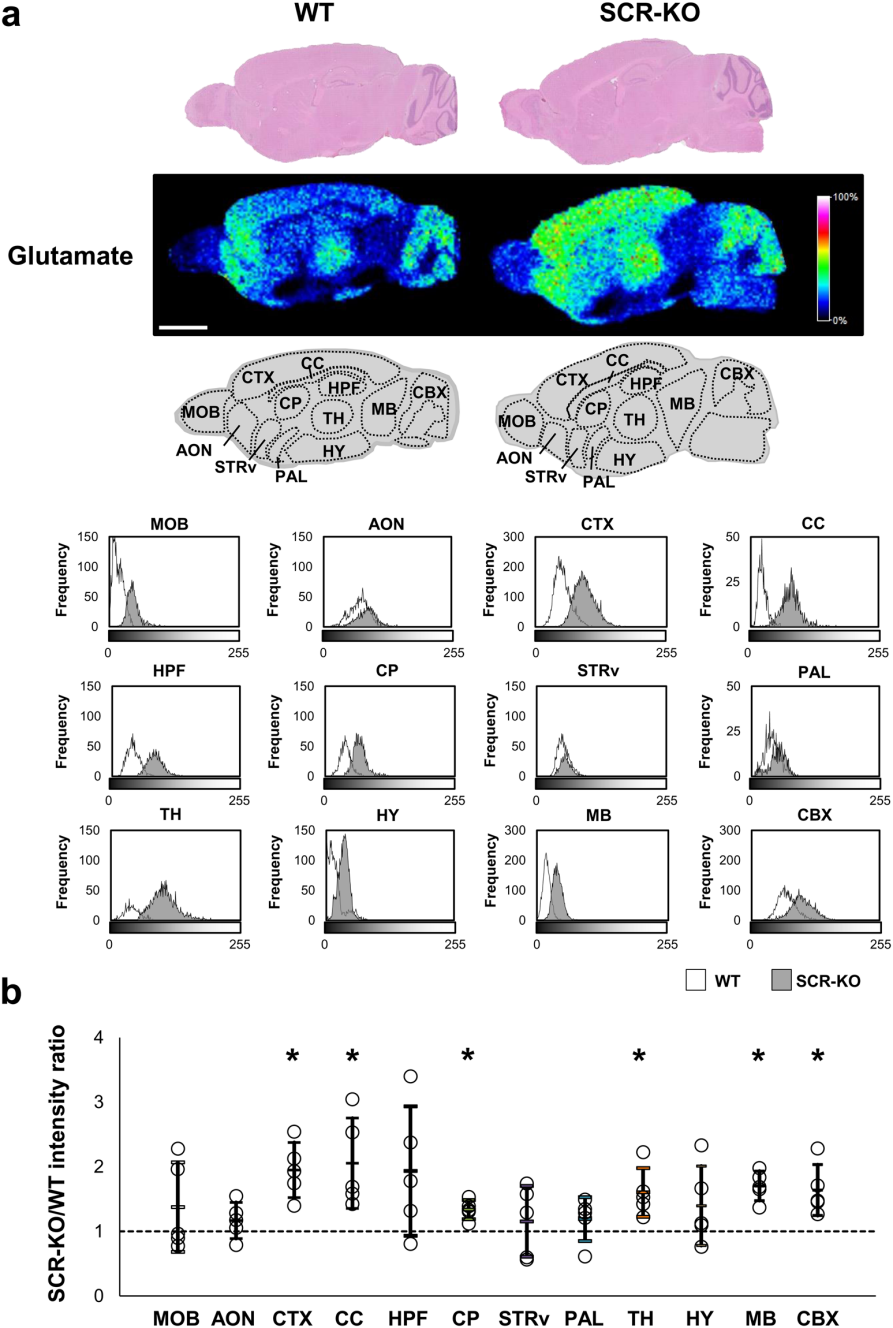
Glutamate increases in several brain regions in *Scrapper*-knockout (SCR-KO) mice. (**a**) Hematoxylin and eosin (HE) stained brain sections of wild type (WT) and SCR-KO mice. Signal intensity of Glu in sagittal brain sections is shown on a rainbow-color scale (scale bar, 2 mm). Several brain regions of WT and SCR-KO mice were selected for detailed analysis and are presented as histograms of grayscale images (scale bar, 2 mm). (**b**) The ratios of the glutamate signal intensity in each brain region between the SCR-KO and WT mice. The SCR-KO/WT intensity ratio of the geometric mean of the signal intensity histogram in each comparison is represented as a point (n = 5 mice); the geometric mean approximates to the peak of the histogram. The arithmetic mean and standard deviation of SCR-KO/WT intensity ratios is displayed as a bar. Asterisks indicate brain regions that featured significant differences (*p < 0.05) in the student’s t test between the SCR-KO/WT intensity ratio and the null distribution, which has a mean of 1 and the same standard deviation as that of the geometric mean distribution of each brain region.

We visualized glutamate in the sagittal brains of SCR-KO and WT mice using IMS. In order to evaluate the distribution of glutamate in each region, we divided the sagittal sections into twelve regions based on the Allen Brain Atlas (http://www.brain-map.org/): main olfactory bulb (MOB), anterior olfactory nucleus (AON), isocortex (CTX), corpus callosum (CC), hippocampal formation (HPF), caudoputamen (CP), striatum ventral region (STRv), Pallidum (PAL), thalamus (TH), hypothalamus (HY), midbrain (MB), and cerebellar cortex (CBX). Subsequently, we used a histogram to show the signal intensity of glutamate of each pixel in each of the brain regions of the SCR-KO and WT mice. Glutamate was more abundant in the AON, CTX, TH, and CBX than in other regions in WT mice but was more abundant in the CTX, CC, HPF, CP, STRv, TH, and CBX than in other regions in SCR-KO mice. Notably, glutamate was less abundant in the HY and MB than in other regions in both SCR-KO and WT mice (Fig. 3a).

We used histograms to compare the amounts of glutamate in various regions of the brain in SCR-KO and WT mice. The horizontal axis of the histograms represents the signal intensity level, and the vertical axis indicates the frequency of pixels. The average, mode, and maximum values of the histograms increased in the MOB, CTX, CC, HPF, CP, TH, MB, and CBX, but not in the AON, STRv, PAL and HY in SCR-KO mice compared with WT mice. Glutamate abundance increased in these brain regions, especially in the CTX, CC, HPF and TH in SCR-KO mice compared with WT mice (Fig. 3a).

Subsequently, for each measurement, the ratio of the geometric mean of the signal intensity in each brain region between SCR-KO and WT mice was calculated, and the differences between the mean values for the null distribution were analyzed. Significant differences (*p* < 0.05) were found in the CTX, CC, CP, TH, MB, and CBX (CTX: p < 0.0000, Cohen’s d = 2.22; CC: p < 0.0004, Cohen’s d = 1.63; CP: p < 0.0000, Cohen’s d = 2.31; TH: p = 0.0009, Cohen’s d = 1.52; MB: p < 0.0000, Cohen’s d = 3.10; CBX: p = 0.0006, Cohen’s d = 1.58). Compared with the glutamate signal intensities of samples obtained from WT mice, those derived from SCR-KO increased by 1.5-fold or more in the CTX, CC, TH, MB, and CBX and by 1.3-fold in the CP (Fig. 3b); however, no significant differences were observed in the MOB, AON, HPF, STRv, PAL and HY (MOB: p = 0.2879, Cohen’s d = 0.48; AON: p = 0.2262, Cohen’s d = 0.55; HPF: p = 0.0595, Cohen’s d = 0.86; STRv: p = 0.4169, Cohen’s d = 0.36; PAL: p = 0.2295, Cohen’s d = 0.55; HY: p = 0.1653, Cohen’s d = 0.63). The glutamate signal intensity was relatively lower in the MB than in other regions in SCR-KO and WT mice. Therefore, the increase of glutamate in each region of the brain in SCR-KO mice does not depend on the amount of glutamate in that region.

### GABA increases in the CTX, CC, TH, HY, MB, and CBX in SCR-KO mice

**Figure 4:**
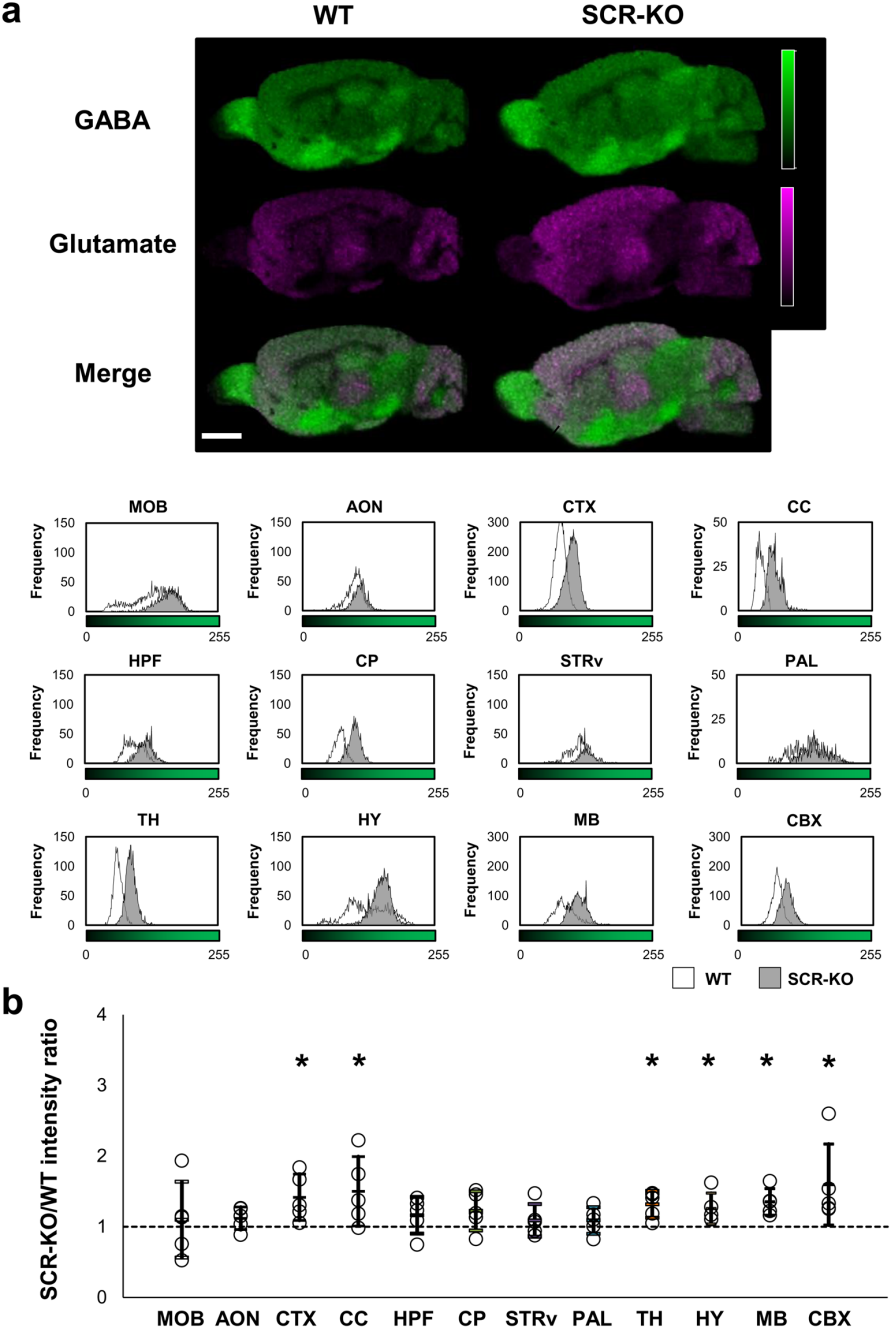
Increases of GABA in the brain regions of SCR-KO mice. (**a**) Signal intensity of GABA in sagittal brain sections is represented on a green-color scale. The merged images of GABA (green) and Glu (magenta) show different distribution patterns of GABA and Glu in the brain sagittal sections. The brightness of the green areas in each brain region of the WT and SCR-KO mice is represented in histograms (scale bar, 2 mm). (**b**) The ratio of GABA signal intensity in each brain region between the SCR-KO and WT mice. As in Fig. 2b, the SCR-KO/WT intensity ratio of the geometric mean of the signal intensity histogram in each comparison is represented as a point (n = 5 mice); the geometric mean approximates to the peak of the histogram. The arithmetic mean and standard deviation of SCR-KO/WT intensity ratios is displayed as a bar. Asterisks indicate brain regions that featured significant differences (*p < 0.05) in the student’s t test between the SCR-KO/WT intensity ratio and the null distribution, which has a mean of 1 and the same standard deviation as that of the geometric mean distribution of each brain region.

In both SCR-KO and WT mice, the signals of GABA were relatively higher in the MOB, PAL, HY, and MB than in other areas. In contrast, as demonstrated above, the glutamate signals were lower in the MOB, PAL, HY, and MB than in other regions (Fig. 4a) in both SCR-KO and WT mice. We made histograms to compare the amount of GABA in each region between SCR-KO and WT mice. The average, mode, and maximum values of the histograms increased in the MOB, AON, CTX, CC, CP, TH, MB, and CBX in SCR-KO mice compared with WT mice (Fig. 4a).

Subsequently, in each measurement, the ratio of the geometric mean of the signal intensity in each brain region between SCR-KO and WT mice was calculated, and the differences between the mean values for the null distribution were assessed. Significant differences (*p* < 0.05) were observed in the CTX, CC, TH, HY, MB and CBX (CTX: *p* = 0.0090, Cohen’s d = 1.19; CC: *p* = 0.0181, Cohen’s d = 1.08; TH: *p* = 0.0002, Cohen’s d = 1.72; HY: *p* = 0.0087, Cohen’s d = 1.20; MB: *p* < 0.0000, Cohen’s d = 1.98; CBX: *p* = 0.0244, Cohen’s d = 1.02). The GABA signal intensity increased by 1.3-fold or more in the CTX, CC, TH, MB and CBX in SCR-KO mice compared with WT mice (Fig. 4b). However, no significant differences were detected in the MOB, AON, HPF, CP, STRv and PAL (MOB: *p* = 0.6150, Cohen’s d = 0.23; AON: *p* = 0.0676, Cohen’s d = 0.83; HPF: *p* = 0.1327, Cohen’s d = 0.68; CP: *p* = 0.1153, Cohen’s d = 0.716; STRv: *p* = 0.2569, Cohen’s d = 0.51; PAL: *p* = 0.2526, Cohen’s d = 0.52).

### Quantitative analyses of glutamate and GABA using LC-MS/MS

Varying concentrations (0.5–250 ng/ml) of standard solutions of GABA and glutamate were plotted to draw the standard curves. The standard curves were generated using least-square linear regression (Glutamate; y = 0.4192x+ 2.3966, r = 0.9999, GABA; y = 0.3535x+ 0.1024, r = 0.9999) (Supplementary Fig. 1). The established LC-MS/MS strategy was applied in the target quantification of glutamate and GABA in the brain of WT and SCR-KO mice (n = 5). Quantitative analyses of glutamate revealed a significant (*p* = 0.0308) increase in the levels of glutamate in the entire brain of SCR-KO mice (2.50 ± 0.42 mmol/L) compared to WT (1.85 ± 0.37 mmol/L). Additionally, GABA also significantly increased (*p* = 0.0493) in SCR-KO mice (0.33 ± 0.06 mmol/L) compare WT (0.25 ± 0.04 mmol/L).

### The number of GFAP-positive cells increases in the CTX, but not in the HPF and TH of SCR-KO mice

**Figure 5:**
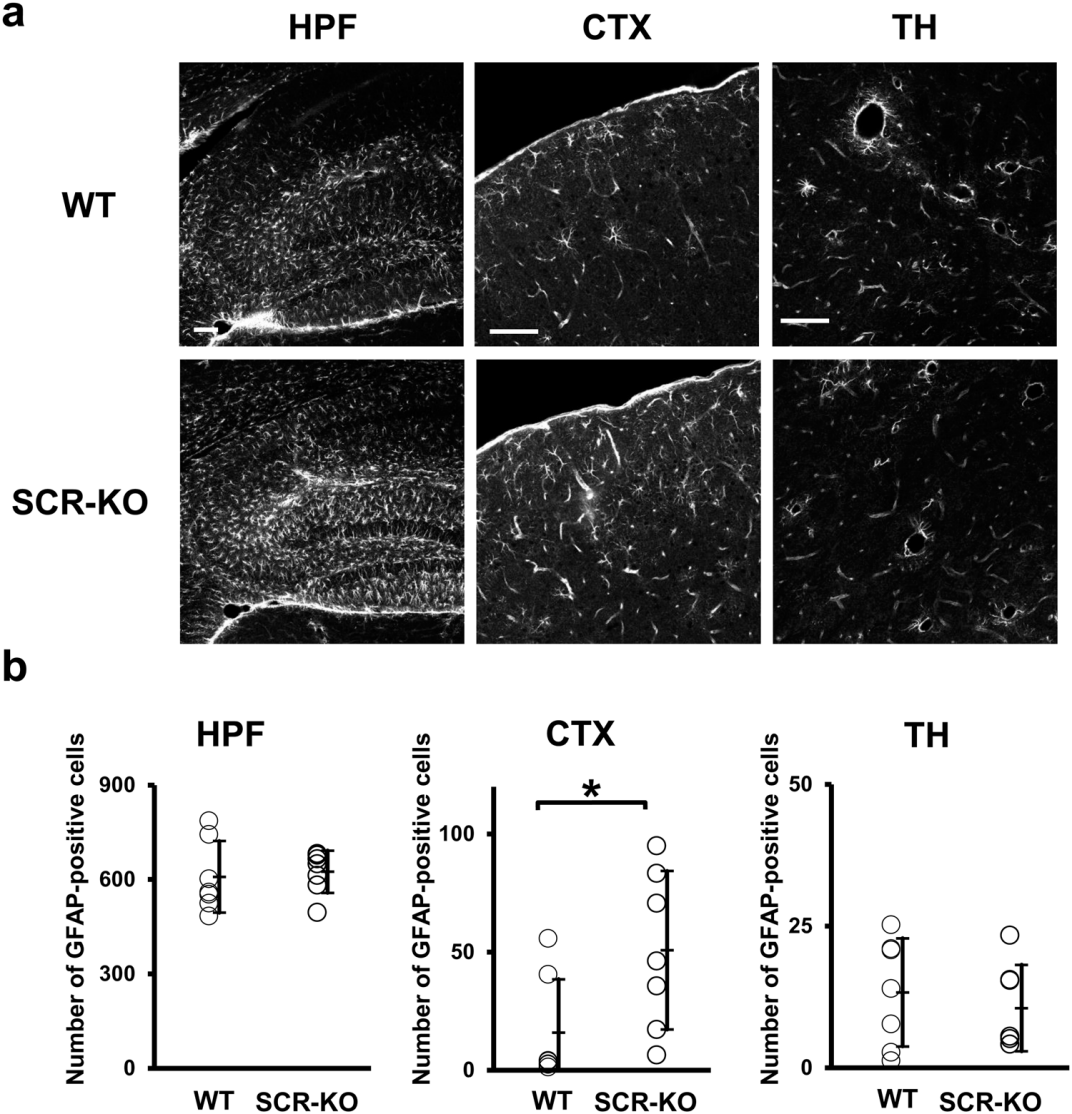
Increases in glial fibrillary acidic protein (GFAP) positive cells in the isocortex (CTX) of SCR-KO mice. (**a**) The HPF, CTX, and thalamus (TH) were stained with primary antibodies against GFAP (scale bar, 100 μm). (**b**) Number of GFAP-positive cells per unit tissue area (mm^2^) was counted and compared in each brain region between WT (n = 7 mice) and SCR-KO mice (n = 7 mice) (* *p* < 0.05).

Astrocytes express the glutamate transporter Slc1a2 in the CTX and HPF^19^, and glutamate transporters have a role in glutamate clearance^20^. It is well-established that glial fibrillary acidic protein (GFAP) plays a key role in modulating astrocytic and neuronal glutamate transporter trafficking^21^. Therefore, an increase in GFAP-positive cells implies increased clearance of glutamate. Here, we aimed to examine whether Scrapper influences glutamate clearance by astrocytes in the CTX, HPF, and TH, where higher intensity signals of glutamate were observed in SCR-KO mice and WT mice in the above-mentioned IMS analyses. The GFAP expression in SCR-KO seemed to increase in the HPF and CTX area (Fig. 5a). We determined the number of astrocytes expressing GFAP in these regions. Significant differences (*p* < 0.05) were found in the CTX of SCR-KO mice compared to WT mice, but not in the HPF and TH (Fig. 5b) (CTX: p = 0.0418, Cohen’s d = 1.22; HPF: p = 0.7517, Cohen’s d = 0.17; TH: p = 0.5595, Cohen’s d = −0.32).

## Discussion

The present study sought to investigate the distribution of glutamate and GABA in the brains of SCR-KO mice. SCR-KO mice showed significantly increased glutamate levels in the CTX, CC, TH, MB, CBX and CP, and enhanced GABA levels in the CTX, CC, TH, MB, CBX and HY. The number of reactive astrocytes increased in the CTX, but not in the HPF and TH in SCR-KO mice compared with WT mice. These results indicate that Scrapper influences the abundance of glutamate and GABA in multiple, specific regions of the brain.

Our previous research using western blot analysis found that Scrapper is almost evenly distributed in the brain^7^. Therefore, we initially hypothesized that *Scrapper* gene deficiency is linked to ubiquitous glutamate upregulation, and thus an excessive secretion of glutamate. However, in the present study, significant differences in glutamate levels were detected in the CTX, CC, TH, MB, CBX, and CP of SCR-KO mice compared to WT mice (Fig. 3b). On the other hand, we found that GABA abundance was significantly increased in the CTX, CC, TH, MB, CBX and HY of SCR-KO mice compared to WT mice (Fig. 4b). Although the CP has high levels of GABAergic projections, GABA signals were not elevated in the CNU of either the SCR-KO or WT mice (Fig. 4a). In previous studies that have relied on LC-MS / MS for quantification, the amount of GABA in the striatum was lower than in the olfactory bulb and hypothalamus, comparable to that in the frontal cortex and cerebellum^22^. Therefore, the striatum is expected to have less GABA abundance than in other regions. The effects of Scrapper on glutamate and GABA abundance were significantly more pronounced in the CTX, CC, TH, MB, and CBX. The fact that both glutamate and GABA are increased in SCR-KO mice indicates that the effects of Scrapper on glutamate levels closely relate to GABA levels. Our quantitative analysis of glutamate and GABA abundance in the whole brain using LC-MS/MS also supports this notion. Collectively, these results suggest that the effects of Scrapper on glutamate and GABA levels vary across brain regions. Our previous results also support the diversity of Scrapper’s effects and showed that some peptide levels in SCR-KO mice were altered in specific regions of the brain^23^.

Subsequently, we revealed the influence of Scrapper on the glutamate clearance of astrocytes expressing GFAP in the CTX, HPF, and TH, where higher intensity signals of glutamate were observed at the tissue level in both SCR-KO and WT mice. We found increased expression levels of GFAP in the CTX, but not in the HPF and TH, of SCR-KO relative to WT mice (Fig. 5). It is well-established that GFAP plays a key role in modulating astrocytic and neuronal glutamate transporter trafficking, and controls glutamine production^21^. In fact, GFAP is involved in the regulation of the glutamate/aspartate transporter (GLAST) on the cell surface^24^. Therefore, we suppose that glutamate clearance is increased in the CTX in SCR-KO mice and that this is modulated by Scrapper. Our previous physiological study^13^, which showed that SCR-KO mice exhibit a longer decay time in spontaneous and evoked EPSCs of the cortex, supports this hypothesis. Additionally, another report showed that activated astrocytes in the cingulate cortex of mice aggravated the manifestation of anxiety-like behavior^25^. Considering that heterozygous SCR-KO mice exhibit reduced fear memory and abnormal social behavior^14^, Scrapper may play an important role in several psychiatric disorders that manifest due to increased neurotransmitters in the anterior cingulate area. In this study, no changes were found in the number of astrocytes expressing GFAP in the HPF and TH of SCR-KO mice compared to WT mice. Furthermore, the MALDI-IMS data analysis indicates that there are no differences in the amount of total HPF glutamate or GABA. A previous report has shown that RIM1 concentrations increased in the hippocampus of SCR-KO mice, and the frequency of miniature EPSC in CA1 of SCR-KO was enhanced^7^. Based on these observations, we suppose that the RIM1-mediated enhanced excitatory glutamatergic transmission of Scrapper does not affect glutamate stocks in the HPF. Therefore, the increase in glutamate caused by Scrapper deficiency may be useful for understanding the mechanisms of anxiety-related disease related to CTX. Further studies constantly measuring *in vivo* concentrations of neurotransmitters in each brain region, such as microdialysis, will help to understand the functional impact of Scrapper on neurotransmission.

Although this study is the first to demonstrate that Scrapper deficiency upregulates glutamate and GABA levels in specific brain regions, the mechanisms underlying this phenomenon have not been examined, limiting any discussions regarding the association between this phenomenon and the expression of Scrapper. Future studies that visualize the distribution of synaptic vesicles and presynaptic molecules using other techniques, such as super-resolution imaging, will help to clarify the brain region-specific functions and mechanisms of Scrapper.

In this study, we found that Scrapper deficiency led to upregulated glutamate and GABA levels in multiple regions of the brain, but led to a selective increase of only glutamate and not GABA levels in the CP, and GABA but not glutamate levels in the HY. In addition, we found that Scrapper-related signaling alters astrocyte expression in the CTX. These findings may shed light on the mechanisms responsible for behavioral abnormalities in mice with an imbalance of neurotransmitter abundance.

## Materials and Methods

### Animals

*Scrapper* knockout (SCR-KO) mice were described previously^7^. These mice were maintained on a mixed C57BL/6 background. Adult (8–16 weeks old) KO and wild type (WT) mice were used in this study. The mice were housed in a standard animal room with a 12/12 h light/dark cycle under standard laboratory chow and water. All experiments were performed in accordance with the guidelines issued by the Institutional Animal Care and Use Committees of Hamamatsu University, School of Medicine. All experiments were conducted with approval from the institutional review board. In this study, we prepared brain sections from individual mice.

### Tissue-section preparation

SCR-KO and WT mice were euthanized by cervical dislocation. Subsequently, the brain tissues were removed quickly and frozen in powdered dry ice. The frozen brain tissues were cut at a thickness of 10 µm for MALDI-imaging and 150 µm for LC-MS/MS using a cryostat microtome (Leica CM1950, Leica Microsystems). We used the brain sections approximately 1 mm in the laterolateral axis with respect to the bregma. In MALDI-imaging, the sagittal brain sections were thaw-mounted on indium tin oxide-coated glass slides (ITO glass, Matsunami Glass Ind., Ltd., Osaka, Japan). After the frozen sections were warmed to room temperature, the glass slides were transferred into a vacuum pump (Diaphragm Type Dry Vacuum Pump DTC-21, Ulvac Kiko, Inc., Miyazaki, Japan) and were left therein for 20 minutes to prevent condensation of the brain sections. In LC-MS/MS, the sagittal brain sections were homogenized and sonicated with 80% acetonitrile solution (50 µL/mg tissue) containing 2% formic acid and the internal standard of glutamate and GABA (l-glutamic acid-^13^C_5_, ^15^N; Sigma-Aldrich, St. Louis, MO, USA; CAS# 202468-31-3, and 4-aminobutyric-2,2,3,3,4, 4-D_6_; C/D/N Isotopes Inc., Pointe-Claire, Quebec, Canada; CAS# 70607-85-1). The homogenates of brain tissue were centrifuged at 20,000 g for 10 min at 4 °C. Supernatants were carefully transferred to tubes and stocked at −20 °C. Before conducting LC-MS/MS, the samples were diluted and any liquid was evaporated to dryness. The samples were then dissolved in 60% acetonitrile and filtered through a 0.2 µL filter. Finally, 5 µL each sample was injected into UPLC-MS/MS for analysis.

### MALDI IMS

The derivatization solution was prepared by dissolving DPP-TFB (Sigma-Aldrich, St. Louis, MO, USA; Cat.# R246875) in methanol (1.33 mg/mL). First, the derivatization solution (approximately 200 µL) was sprayed manually and applied to each section. Then, 40 mg/mL 2,5-dihydroxybenzoic acid (Bruker Daltonics) in 50% methanol was uniformly sprayed over the samples with an automatic sprayer (TM-Sprayer, HTX Technologies). MALDI IMS was performed using Fourier-transform ion cyclotron resonance mass spectrometer (Solarix XR 7.0 T, Bruker Daltonics). Our set laser focus was set to “small”, laser pulses were 1000 Hz, and the number of irradiations was 200 shots. The raster step size was set to 100 µm. We obtained positive ions in a mass range of *m/z* 100.33 to 1000. Subsequently, the sections were used for hematoxylin and eosin (HE) staining. Imaging data were analyzed using fleximaging software (Bruker Daltonics). The images were used to analyze the distribution of glutamate and GABA using an image-analysis software (ImageJ; https://imagej.nih.gov/ij/).

### LC-MS/MS

Standards of glutamate and GABA utilized for construction of calibration curves were diluted to the proper concentrations with 60% acetonitrile. Standard curves were prepared by adding different amounts of analytes to 60% acetonitrile to produce final concentrations ranging 0.5–250 ng/mL for GABA and glutamate, respectively. A constant amount of internal standards were added to all standard solutions to produce a fixed concentration. LC-MS/MS was performed with an Acquity UPLC system (Waters, USA) an autosampler and a 4000 QTRAP linear ion trap quadrupole mass spectrometry system (Sciex, Foster City, CA, USA). All operations, including the acquiring and analysis of data, were controlled by Analyst software (version 1.6.1, Sciex). The mobile phase consisting of 0.1% formic acid (A) and acetonitrile (B) was delivered at a flow rate of 0.2 mL/min. The linear gradient elution program was as follows: 0–1 min, 1% A; 1–7 min, 1–70% A; 7–9 min, 70% A; 9–9.1 min, 70–1% A; 9.1–15 min, 1% A. The injection volume was 5 µL and the total time taken for the chromatographic run was 15 min. The analytes were ionized by the ESI source in positive ion mode.

### Immunohistochemistry

Mice were administered anesthetics, consisting of medetomidine (0.75 mg/kg), midazolam (4.0 mg/kg), and butorphanol (5.0 mg/kg), by intraperitoneal injection. Mice were then transcardially perfusion-fixed with 4% paraformaldehyde phosphate buffer solution. Harvested brains were post-fixed overnight in the fixing solution and were then put in 30% sucrose for cryoprotection. The frozen brain tissues were cut at a thickness of 20 µm using a cryostat microtome. We used brain sections approximately 1 mm in the lateral axis with respect to the bregma. The free-floating sections were incubated in an appropriate blocking solution and then with anti-GFAP antibodies (Sigma-Aldrich; Cat.# G3893, RRID:AB_477010) (1:2000 dilution in phosphate-buffered saline/0.3% bovine serum albumin and 0.025% Triton-X). After being washed thrice, the sections were incubated with appropriate secondary antibodies conjugated with Goat Anti-Mouse IgG (H + L) Highly Cross-adsorbed Antibody, Alexa Fluor 568 Conjugated (Molecular Probes; Cat.# A-11031, RRID:AB_144696) (1:1000 dilution in phosphate-buffered saline/0.3% bovine serum albumin and 0.025% Triton-X). Fluorescence of immunolabeling was detected using a confocal microscope (Leica TCS SP8, Leica microsystems, Wetzlar, Germany). The images were used to analyze the distribution of GFAP using an image analysis software (ImageJ; https://imagej.nih.gov/ij/). We reviewed the shape of each image of the astrocytes and determined the threshold intensity of conversion to capture clear images. Then, the number of GFAP-positive cells was counted in each tissue area. Cell counts are reported as number of GFAP-positive cells per unit tissue area (mm^2^).

### Statistical analysis

To compare signal intensities of glutamate and GABA between SCR-KO and WT mice, we first acquired data on the intensity of each pixel of the image, which was separated into twelve brain regions. We then calculated the geometric mean of the data because histograms of signal intensity at any *m/z* in each pixel were empirically approximated to log-normal distribution rather than the normal distribution. After that, we calculated the ratio of these geometric means as SCR-KO/WT and repeated this process for the additional five biological replicates. In IMS data, a fluctuation between measurements can disrupt the analysis of biological samples. To detect differences between SCR-KO and WT mice from the data, we put one sample each of SCR-KO and WT mice on one slide and calculated the ratio. Four biological replicates were continuously measured, and the ratios were calculated. Next, arithmetic means of the four ratios of geometric means as SCR-KO/WT and standard deviations were calculated. Distribution of these ratios was hypothesized as Gaussian by the central limit theorem. Therefore, subsequently, we generated 200 random numbers, which follow the Gaussian distribution, as null distribution using 1 as mean and standard deviation calculated from the five biological replicates in the previous step. Finally, we performed the student’s t-test and calculated the effect size (Cohen’s d) between the five ratios of geometric means observed from tissues and the 200 generated numbers (null distribution). In the LC-MS/MS and immunohistochemistry analysis, statistical comparisons were made using student’s t-test. All data are presented as mean ± standard deviation (S.D.). In all case, *p* < 0.05 was considered statistically significant.

## Supplementary information

Supplementary Information.

## Data Availability

The datasets generated during and/or analyzed during the current study are available from the corresponding author on reasonable request.
