# Supplementary material for: Region-specific effects of Scrapper on the abundance of glutamate and gamma-aminobutyric acid in the mouse brain
Source: Sci Rep. 2020 May 4;10:7435. doi: 10.1038/s41598-020-64277-w (PMC7198594; doi:10.1038/s41598-020-64277-w)
Supplement: Supplementary file 1 — Supplementary Information. [file 41598_2020_64277_MOESM1_ESM.pdf]

Supplementary Information to

Region-specific effects of Scrapper on the abundance of glutamate and gamma-aminobutyric acid in the mouse brain

Fumihiro Eto[1,2,3], Shumpei Sato[2,4], Mitsutoshi Setou[2,4,5], and Ikuko Yao[1,2,3,4 *]

[1]Department of Optical Imaging, Institute for Medical Photonics Research, Preeminent Medical Photonics Education & Research Center, Hamamatsu University School of Medicine, 1-20-1 Handayama, Higashi-ku, Hamamatsu, Shizuoka 431-3192, Japan

[2]Department of Cellular and Molecular Anatomy, Hamamatsu University School of Medicine, 1-20-1 Handayama, Higashi-ku, Hamamatsu, Shizuoka 431-3192, Japan

[3]Department of Biomedical Chemistry, School of Science and Technology, Kwansei Gakuin University, 2-1 Gakuen, Sanda, Hyogo 669-1337 Japan

[4]International Mass Imaging Center, Hamamatsu University School of Medicine, 1-20-1 Handayama, Higashi-ku, Hamamatsu, Shizuoka 431-3192, Japan

[5]Department of Systems Molecular Anatomy, Institute for Medical Photonics Research, Preeminent Medical Photonics Education & Research Center, Hamamatsu University School of Medicine, 1-20-1 Handayama, Higashi-ku, Hamamatsu, Shizuoka 431-3192, Japan

[*] **Correspondence author's name and email address:**

Ikuko Yao, Ph.D.

E-mail: yaoik@kwansei.ac.jp

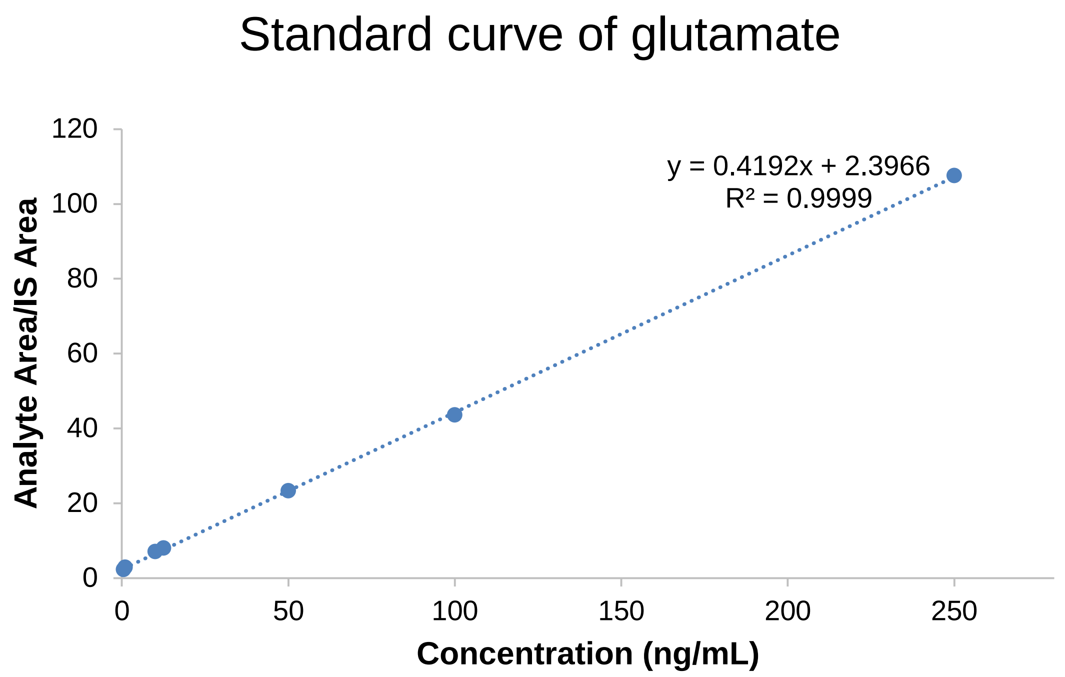

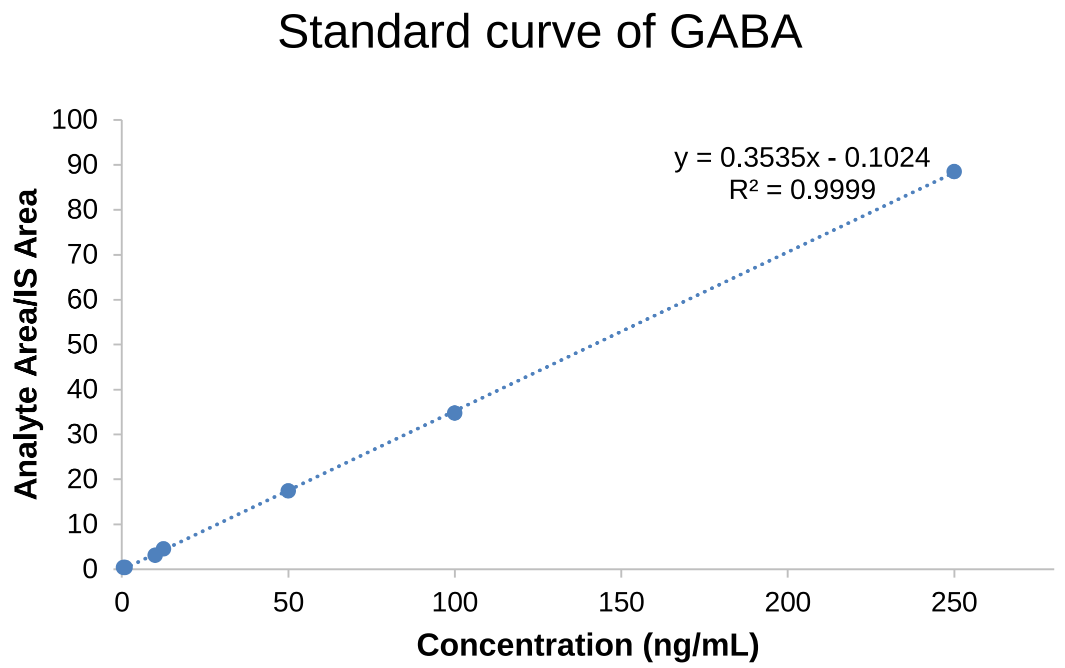

Supplementary fig. 1 Standard curve of glutamate and GABA in the LC/MS analysis Varying concentrations (0.5–250 ng/ml) of standard solutions of GABA and glutamate were plotted to draw the standard curves. The standard curves were generated using least-square linear regression (Glutamate; $y = 0.4192x + 2.3966$, $r = 0.9999$, GABA; $y = 0.3535x + 0.1024$, $r = 0.9999$).
